# The relationship between motherhood and use of mental health care services among married migrant and non-migrant women: a national register study

**DOI:** 10.1186/s12888-022-03848-9

**Published:** 2022-03-21

**Authors:** Melanie Straiton, Anna-Clara Hollander, Kamila Angelika Hynek, Aart C. Liefbroer, Lars Johan Hauge

**Affiliations:** 1 Department of Mental Health and Suicide, Norwegian Institute of Public Health, PO Box 222, Skøyen, 0213 Oslo, Norway; 2 Epidemiology of Psychiatric Conditions, Substance Use and Social Environment, Department of Global Public Health, Karolinska Institute, 171 77 Stockholm, Sweden; 3 Netherlands Interdisciplinary Demographic Institute, PO Box 11650, 2502 AR The Hague, The Netherlands; 4 Department of Epidemiology, University Medical Centre Groningen, University of Groningen, Groningen, The Netherlands; 5 Department of Sociology, Vrije Universiteit, Amsterdam, The Netherlands

**Keywords:** motherhood, mental disorder, post-partum disorder, migrants, health service use

## Abstract

**Background:**

Giving birth to one’s first child is a life changing event. Beyond the post-partum period, little is known about the association between becoming a mother and mental disorder among migrant women. This study investigates outpatient mental health (OPMH) service use, a proxy for mental disorder, among married migrant and non-migrant women who become mothers and those who do not.

**Methods:**

Using Norwegian register data, we followed 90,195 married women, aged 18-40 years, with no children at baseline between 2008-2013 to see if becoming a mother was associated with OPMH service use. Data were analysed using discrete time analyses.

**Results:**

We found an interaction between motherhood and migrant category. Married non-migrant mothers, both in the perinatal period and beyond, had lower odds of OPMH use than married non-mothers. There was no association between motherhood and OPMH service use for migrants. However, there was no significant interaction between motherhood and migrant category when we excluded women who had been in Norway less than five years. Among women aged 25-40 years, a stable labour market attachment was associated with lower odds of OPMH use for non-migrants but not migrants, regardless of motherhood status.

**Conclusions:**

The perinatal period is not associated with increased odds of OPMH use and appears to be associated with lower odds for married non-migrant women. Selection effects and barriers to care may explain the lack of difference in OPMH service use that we found across motherhood status and labour market attachment for married migrant women. Married migrant women in general have a lower level of OPMH use than married non-migrants. Married migrant women with less than five years in Norway and those with no/weak labour market attachment may experience the greatest barriers to care. Further research to bridge the gap between need for, and use of, mental health care among migrant women is required.

**Supplementary Information:**

The online version contains supplementary material available at 10.1186/s12888-022-03848-9.

## Background

The transition to motherhood is a major life-event. For many, the perinatal period can be a time of joy [1]. However, it is also considered a sensitive time period for the onset of mental disorders [2]. Post-partum depression is a common mental disorder thought to affect around 17% of new mothers globally, though estimates vary from 3-38% [3, 4]. In addition to physiological and hormonal changes associated with pregnancy and childbirth, poor psychosocial adjustment to motherhood can, over time, have an impact on the mental health of mothers [5, 6]. It is unclear however, if mothers, both in and beyond the perinatal period are at higher or lower risk of mental disorder than women who voluntarily or involuntarily have no children [7]. Further, research from high income countries indicates that migrant women, particularly those from countries outside of the European Economic Area (EEA), the UK, USA, Canada, Australia and New Zealand, may be at higher risk of post-partum depressive symptoms than non-migrant women [8, 9]. It is, however, not known if these differences are pre-existing or due to the transition to motherhood.

### Motherhood and mental health

While cross-sectional studies usually indicate that mothers report better mental health, well-being and life-satisfaction than women who voluntarily or involuntarily have no children [10–12], a longitudinal study indicated that mother’s well-being may decrease over time to a greater extent than non-mothers [13]. However, direct comparison of findings is difficult given the different study designs, definitions of mothers, the inclusion or exclusion of non-mothers or mothers with older children as controls and different ways of measuring mental health [7]. There are also few studies comparing the risk of *mental disorder* among mothers compared with non-mothers beyond the post-partum period. Self-reported studies in Germany and Australia found no difference in the rates of current or life-time depression or other mental disorders between mothers and non-mothers [7, 12]. These studies were, however, cross-sectional and were unable to account for mental health selection into motherhood.

The association between motherhood and mental disorder may also depend on sociodemographic factors. For instance, younger mothers appear to be at greater risk of mental disorder [14]. In a longitudinal Danish register study, mothers aged 26 or less, had a higher risk of mental disorder, measured by hospital admission and outpatient contact with mental health services, compared with women without children [15]. In contrast, mothers aged 30 or over, had a lower risk than women without children. Civil status and employment can also play a role. Single mothers are more likely to report major depression than married mothers [6, 16] and employed mothers may experience less parenting strain and better mental health than non-employed mothers [6, 17]. Further, a study across 28 European countries found that in countries with more extensive childcare provision, such as in the Nordic countries, mothers tend to report more happiness than non-mothers, whereas in countries with poorer provision, mothers report less happiness [18]. This suggests that the social and political context plays an important role in whether mothers experience better mental health than women who voluntarily or involuntarily have no children.

One notably absent aspect from the limited literature on mental disorder among mothers and women without children is attention to migrant women. Although some research indicates that migrants are at greater risk of post-partum depression than their non-migrant counterparts [19, 20], we know little about the relationship between motherhood and mental disorder beyond the perinatal period for migrants. Additionally, it is possible that the relationship differs for different groups of migrant women. We know that general differences between mothers and women who voluntarily or involuntarily do not have children may be time and context specific [21]. For instance, in many parts of Europe, it is no longer considered a tragedy or failure not to become a mother [22]. Lower stigma overtime may lead to better mental health for women who voluntarily or involuntarily have no children. However, this may not apply to all groups of women within a society. Childlessness for instance, may be stigmatised among Pakistani women in the UK [23] and it threatens the social status of Zimbabwean women in Australia [24]. In the Netherlands, Turkish migrant women report more social pressure to have children, and more emotional distress if they are unable to, than their Dutch counterparts [25]. Thus, if childbearing is more important for migrants from countries with other predominating norms (for example, in Asia, Africa, Latin America, Oceania (except for Australia & New Zealand) and European countries not in the EEA or the UK), the mental health difference between migrant mothers and migrant women without children could be larger than for non-migrant women.

On the other hand, migrant mothers in general, without family in their country of residence may be less likely to receive help and support from family with childcare [26, 27]. Some may feel conflicted by different cultural child-rearing practices [26, 27], experience discrimination or social exclusion from non-migrant mothers [28]. Some migrant women may also struggle as a mother in the public sphere due to language difficulties or unfamiliarity with the country’s system such as the organisation of childcare or school systems [29]. If motherhood is a greater source of stress for migrant women, the association between motherhood and mental disorder could be different for migrant women relative to non-migrant women.

Yet another aspect of motherhood that may differ for migrant women, particularly those from countries where men are predominantly the breadwinner, is that of combining motherhood with employment. Workforce participation is usually associated with better mental health, partly due to an increase in socioeconomic resources for the family [17]. However, in the general population, an unequal division of labour and more work-family conflict is related to higher levels of stress and more sick-leave [30–32]. Partners’ involvement in childcare and household tasks may be important for women’s wellbeing, especially in the early years of childhood. Father’s involvement in housework and childcare has increased substantially in recent decades in many European, especially Nordic, countries [33]. However, migrant women from countries with a more traditional gender division of labour, may still have more responsibility for childcare and housework relative to their non-migrant counterparts, regardless of their employment status [34–37]. The ‘double shift’ may thus be longer for many mothers from for instance, Asia, Africa, Latin America, Oceania (except for Australia & New Zealand) and European countries not in the EEA or the UK, resulting in higher levels of stress. Further, migrant women from these countries may be more likely to work in physically demanding jobs and have less control over their working schedule than non-migrant women [38]. Greater control is associated with better well-being among parents [39]. It is therefore possible that working outside the home could be less protective for mothers from Africa, Asia, Latin America, Oceania (except for Australia & New Zealand) and European countries not in the EEA or the UK than for non-migrant mothers or mothers from for instance, countries in the EEA, the UK, USA, Canada, Australia or New Zealand.

In summary, there are potentially several reasons for why motherhood could relate to mental disorders for migrant and non-migrant women differently. This could also differ somewhat for migrant women from different parts of the world. Workforce participation may also play an important role.

### Current study

In the current longitudinal study, we focused on married women living in Norway, aged 18-40 years, who voluntarily or involuntarily have no children at baseline and no recent history of outpatient mental health (OPMH) service use and followed them for up to six years. We aimed to answer the following research questions: 1) Do married women who become mothers have higher or lower odds of using OPMH services (a proxy for mental disorder) compared with married women who do not? 2) Does the relationship between motherhood and OPMH service use differ for married migrant women compared to married non-migrant women? 3) Does the relationship between workforce participation and mental disorder differ for married migrants from Asia, Africa, Latin America, Oceania (except for Australia & New Zealand) and European countries not in, the EEA or the UK compared with married non-migrants and is this dependent on motherhood status?

## Method

### Setting of the study - migrant women and healthcare in Norway

Migrants (those born abroad with two foreign born parents) in Norway now make up 15% of the population [40]. Just less than half of migrants are from other European countries, around one third from Asia and 14% from Africa. Women represent around 48% of migrants. Half of migrant women come to Norway for family reunification, one in five for work, 16% for protection and 13% to study. They are very diverse in terms of education and employment. Women from EEA countries and the UK tend to have a higher level of employment and are more likely to have higher education than women from other countries [41, 42].

Health care in Norway is a publicly funded universal system and is available to all long-term (over six months) residents and registered asylum-seekers. It is divided into two main sectors: primary (including general practitioners (GPs), emergency care and long-term services) and secondary (hospital and specialised services). Residents are assigned a GP who is the gatekeeper to specialised services, including OPMH services. OPMH services are local specialised services where those with acute mental health problems or who need long-term follow-up can receive help. A referral from a doctor or psychologist is required.

### Data sources

We used data from several national databases and registries, linked at an individual level through a non-identifiable version of a personal number. All registered residents with at least six months of residence are assigned this personal number, in addition to Norwegian-born individuals. Demographic information was extracted from the National Population Registry. This was used to identify all women, their year of birth, civil status, migrant status / region of origin, year of migration and year of any childbirths. Education level was extracted from the Education Database. FD-Trygd, which contains event history information relating to welfare benefits, was used to extract information on child benefit. Information on income was taken from the National Income Tax Registry. Finally, the National Database for the Reimbursement of Health Expenses, which contains information about patient contacts, was used to extract information on whether an individual had attended OPMH and primary health care services.

### Study population

**Fig. 1:**
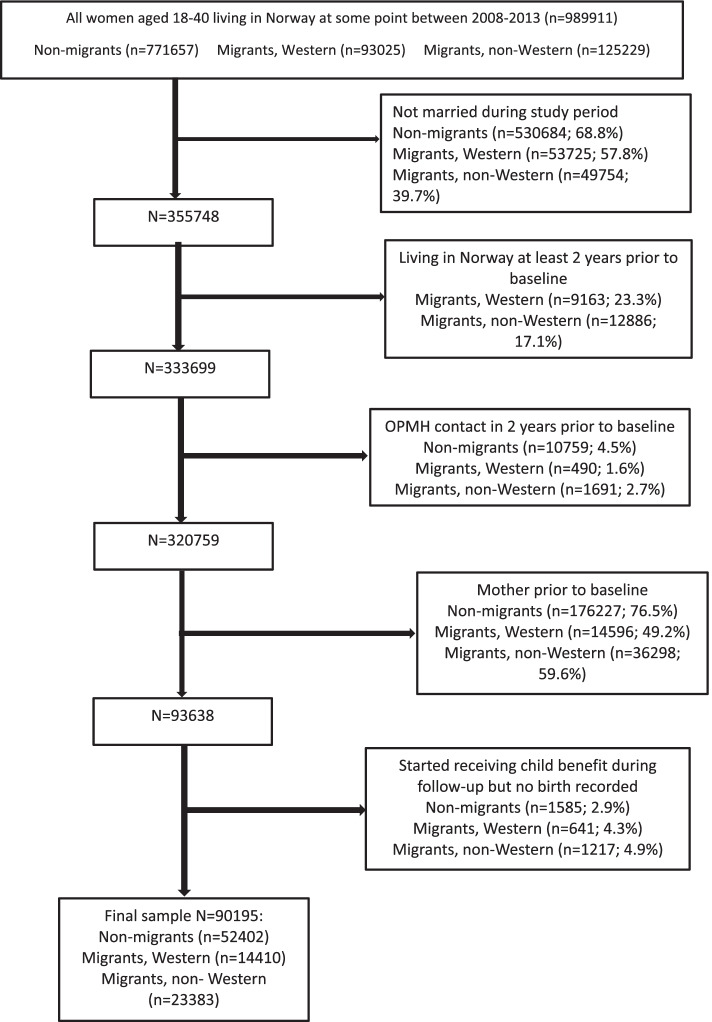
Flow chart with sample inclusion criteria by migrant category

We used a dynamic study design where all women, aged 18-40 years, living in Norway between 2008 and 2013 were potentially included. We selected all women who were married at some point during the study period. Migrant women had to have been living in Norway for at least two years prior to inclusion. This was because we wanted to exclude women who had used OPMH services in the two years prior to baseline to increase the likelihood that use of OPMH services was related to the perinatal period and not a pre-existing condition. Information on women’s year of childbirth (available from 1992) and receipt of child benefit (available from 1996) was used to exclude women who had children prior to baseline (baseline was 2008 or the year of marriage, whichever came first). Women who, during the follow-up period, received child benefit but had not been registered as giving birth were also excluded from analysis because it was unclear when they became mothers. Figure 1 shows a flow chart of the number of women in the study population and the number and percentage of women excluded according to the different inclusion criteria by migrant category.

We included only married women due to lack of cohabiting information for unmarried women. Over 80% of unmarried women in Norway who give birth live with a partner [43]. Thus, most unmarried women who become mothers have a partner, while among unmarried women who do not have children, there is a greater mix of single and cohabiting women. Since cohabitation is almost as protective of mental disorder as having a spouse [44], if we included unmarried women, we would not be able to separate the association between motherhood and OPMH service use and having a partner and OPMH service use among unmarried women. This could lead to an overestimation of any positive effect of giving birth to a child or cancel out any negative effect. Thus, we selected only married women since their partner status was known.

However, because the rate and timing of marriage and giving birth is different across migrant status, selecting only married women results in an overrepresentation of migrant women. As Fig. 1 shows, only 7% (52,402/771,657) of all non-migrant women from the base sample are included in the analysis, as opposed to 15% (14,410/93,025) of all Western migrant women, and 19% (23,383/125,229) of all non-Western migrant women. We consider this selection issue further in the discussion. We followed the women from baseline until they had an OPMH consultation or were censored at the end of 2013, the year they turned 40, died or emigrated. We also censored women if their marital status changed.

### Variables

Outcome: Consultation with OPMH services (yes/no).

Exposure: Motherhood: (Non-mother; mothers, perinatal; mothers, >perinatal). This measure was based on registered births in the medical birth registry. This measure was time-varying in that women were classed as non-mothers until the year they gave birth. Since the perinatal period is considered a vulnerable time for mental disorder development, we also grouped women who were perinatal (year of, or year after birth) separately from mothers who gave birth more than two calendar years ago. Although the post-partum period is generally considered up to 12 months [45], we did not have information on the date of the birth within the calendar year. Thus, inclusion of the following year allowed us to account for the post-partum period for women giving birth towards the end of the year. For example, a woman who gives birth in 2010 would be classed as perinatal in 2010 and 2011. In 2012, she would be classed as >perinatal.

Migrant category: Non-migrant women (women born in Norway and women born abroad with at least one Norwegian born parent) and migrant women (born abroad with two foreign-born parents). We divided migrant women into two categories: Western (women from EEA countries, the UK, USA, Canada, Australia or New Zealand) and non-Western (Asia, Africa, Latin America, Oceania (except for Australia & New Zealand) and European countries not in the EEA or the UK). Country composition is shown in Supplementary table 1. A more nuanced distinction by region of origin was not possible due to power considerations (based on the relative infrequency of the outcome).

Age group (time-varying): Based on year of birth, we grouped women into the following categories: 18-24 years, 25-29 years, 30-34 years, 35-40 years.

Education (time-varying): Highest level of completed education: Higher education, high school education or less than high school education/unknown.

Frequent primary healthcare attendance: This is a proxy of poor somatic health as it is associated with pre-existing medical conditions, unresolved health complaints, medication use and poorer self-reported physical health [46, 47]. Frequent attendance can be defined as the top 10^th^ percentile of number of appointments [48]. We calculated the average number of consultations in primary care across the two years prior to baseline for reasons not related to pregnancy or family planning or for psychological consultations (W and P chapters of ICPC-2 codes respectively). Around 90% of women had an average of four or fewer appointments in the two years prior to baseline. Thus, we had a dichotomous variable of non-frequent attenders (<=4 appointments) and frequent attenders (> 4 appointments).

Labour market attachment (time-varying): (Stable/No or weak) Women with a personal income (based on salary and income from self-employment) of over two times the yearly basic taxation amount of the National Insurance Scheme [49] were classed as participating in the workforce. This cut-off has been used as an indicator of stable labour market attachment in other studies [50].

Ongoing education (time-varying): (Yes/No) Women enrolled in education were classified as currently studying.

Employed/studying (time-varying): (Yes / No). Since younger women are less likely to have entered the workforce because they are still in education, we combined labour market attachment with current enrolment in education. Women who were working and/or studying were grouped together as were women who had a weak or no labour market attachment and were not studying.

### Statistical analyses

Data were arranged on a yearly basis. In the descriptive analyses, we divided and described the sample by migrant category based on the final year of inclusion. We conducted chi-square analyses to see if there was a significant difference across the different groups on each variable. For the main analyses, we conducted discrete-time logistic regression to examine the relationship between becoming a mother and use of OPMH services [51]. First, we ran separate bivariate analyses for each variable, then in model one we included motherhood and migrant category together. In the second model, we controlled for age, education and frequent primary healthcare attendance and in the third model, we added in employed/studying. To investigate if the relationship between motherhood and OPMH service use was moderated by migrant category, we introduced an interaction term between migrant category and motherhood while controlling for all covariates. We ran post-hoc margin analyses and plotted marginal yearly probabilities to visualise the interaction effects. To check the robustness of our findings, we reconducted analyses including only migrants who had been in Norway for at least five years at baseline (length of stay= current year – year of arrival). For this, we selected the sample from the study population in the same way as shown in Fig. 1, except this time, the second exclusion criterium was increased to a minimum of five years in Norway.

Finally, to investigate if the relationship between labour market attachment and mental disorder differed for migrants compared to non-migrants and if it was dependent on motherhood status, we also conducted analyses with 25-40 year olds only, since many in the youngest age category would still be studying. Here, we ran a fully-adjusted model with no interaction, a model with an interaction term between migrant category and motherhood status, a model with an interaction term between migrant category and labour market attachment and then a three-way interaction term between migrant category, labour market attachment and motherhood. We then ran post-hoc margin analyses and plotted marginal yearly probabilities for the final model to visualise the interaction effects. Finally, we reran the analyses again including only migrants who had been in Norway for at least five years.

## Results

### Population sample

Our total population sample consisted of 90,195 married women with 317,665 person years. Women were in the study for a mean of 3.52 years (range: 1-6). Around 58% were non-migrant women. Migrant women are overrepresented compared to the share of migrants in the general population because non-migrant women are less likely to be married and are more likely to have children prior to baseline (see Fig. 1). Overall, half of women became mothers during the follow-up period and four percent used OPMH services. In the sample including only migrants with five or more years of residency in Norway at baseline, non-migrants made up 72% of the sample, half became mothers and overall, 5% used OPMH services. Overall, the average age of becoming a mother was 29.75 years.

Table 1 displays the demographics of the sample by migrant category in the last year of inclusion, both for the total sample and in the sample excluding migrants with less than five years of residency in Norway. There were significant differences by migrant category across all variables.

**Table 1:** Characteristics of the sample by migrant category (% by migrant category) ^a^

	Total sample				Migrants > 5 years in Norway at baseline		
	Non-migrants (*N*=52402)	Migrants, Western (*N*=14410)	Migrants, non-Western (*N*=23383)	Total sample (*N*=90195)	Migrants, Western (*N*=6895)	Migrants, non-Western (*N*=13701)	Total with non-migrants (*N*=72998)
Mean (sd) years in study	3.82 (1.86)	2.93 (1.68)	3.21 (1.82)	3.52 (1.86)	4.16 (1.53)	4.24 (1.65)	3.93 (1.80)
Motherhood							
Non-mother	22137 (42.24%)	8882 (61.64%)	13835 (59.17)	44854 (49.73%)	3042 (46.99%)	6556 (47.85%)	31933 (43.75%)
Mother, perinatal	10497 (20.03%)	2639 (18.31%)	4053 (17.33%)	17189 (19.06%)	1062 (15.40%)	2106 (15.37%)	13665 (18.72%)
Mother, > perinatal	19768 (37.72%)	2889 (18.31%)	5495 (23.50%)	28152 (31.21%)	2593 (37.61%)	5039 (36.78%)	27400 (37.54%)
Used OPMH services	2717 (5.18%)	354 (2.61%)	794 (3.28%)	3864 (4.29%)	300 (4.35%)	619 (4.52%)	3636 (4.98%)
Age group							
18-24 years	3822 (7.29%)	421 (2.92%)	1583 (6.77%)	5826 (6.46%)	105 (1.52%)	796 (5.81%)	4723 (6.47%)
25-29 years	14174 (27.05%)	3330 (23.11%)	6451 (27.59%)	23955 (36.56%)	1030 (14.95%)	3408 (24.87%)	18612 (25.50%)
30-34 years	18641 (35.57%)	5162 (35.82%)	8021 (34.30%)	31824 (35.28%)	2766 (40.12%)	4779 (34.88%)	26186 (35.87%)
35-40 years	15765 (30.08%)	5497 (38.15%)	7328 (31.34%)	28590 (31.70%)	2994 (43.42%)	4718 (34.44%)	23477 (32.16%)
Education							
<high school/unknown	4790 (9.14%)	4352 (30.20%)	10491 (44.87%)	19633 (21.77%)	1670 (24.22%)	5490 (40.07%)	11950 (16.37%)
High school	11751 (22.42%)	2984 (20.71%)	3942 (16.86%)	18677 (20.71%)	1398 (20.28%)	2763 (20.17%)	15912 (21.80%)
Higher	35861 (68.43%)	7074 (49.09%)	8950 (38.28%)	51885 (57.53%)	3827 (55.50%)	5448 (39.78%)	45136 (61.83%)
Employed/studying							
No	6731 (12.84%)	5237 (36.34%)	9875 (42.23%)	21843 (24.22%)	1768 (25.64%)	4238 (30.93%)	12737 (17.45%)
Yes	45671 (87.16%)	9173 (63.66%)	13508 (57.77%)	68352 (75.78%)	5127 (74.36%)	9463 (69.07%)	60261 (82.55%)
Frequent primary care attendence	6524 (12.45%)	637 (4.42%)	1887 (8.07%)	9048 (10.03%)	433 (6.28%)	1418 (10.35%)	8375 (11.47%)
Mean (sd) age of becoming a mother	29.72 (4.05)	30.52 (3.88)	29.39 (4.44)	29.75 (4.13)	30.80 (3.77)	29.32 (4.38)	29.75 (4.10)

^a^ the last year of inclusion is used for the time-varying variables

### Motherhood and OPMH service use

Table 2 shows the yearly odds of using OPMH services during follow-up for the different models. The first column shows the bivariate relationship between OPMH services and each of the variables separately. Married mothers, both in and beyond the perinatal period, had significantly lower odds of using OPMH services than married non-mothers, as did both groups of married migrant women compared with married non-migrant women. Women aged 35-40 years had lower yearly odds than women aged 25-29 years while women aged 18-24 had higher odds. Women who were not employed/studying and women without higher education had higher yearly odds of using OPMH services than those who were employed/studying or had higher education respectively. Frequent attenders of primary healthcare had around three times the yearly odds of women who were not frequent attenders. With both motherhood and migrant category in the model, both groups of mothers had lower yearly odds of OPMH services than non-mothers (model 1). After adjusting for age group, education and frequent attendance (model 2), perinatal mothers in the perinatal period, but not beyond, had lower odds of OPMH service use compared to non-mothers. This was only a tendency after adjustment for whether a woman was employed/studying (model 3).

**Table 2:** Odds ratios and 95% confidence intervals for outpatient mental health service use– all married women

	Bivariate analyses (*n*=90195)	Model 1 (*n*=90195)	Model 2 (*n*=90195)	Model 3 (*n*=90195)	Model 4 (*n*=90195)
Non-mother	1.00	1.00	1.00	1.00	1.00
Mother, perinatal	0.91 (0.85-0.99)*	0.88 (0.82-0.95)**	0.92 (0.85-0.99)*	0.93 (0.86-1.01)^	0.89 (0.81-0.98)*
Mother, > perinatal	0.90 (0.82-0.97)**	0.85 (0.78-0.93)***	0.94 (0.86-1.03)	0.94 (0.86-1.04)	0.88 (0.80-0.97)*
Non-migrants	1.00	1.00	1.00	1.00	1.00
Migrants, Western	0.69 (0.62-0.77)***	0.68 (0.61-0.75)***	0.71 (0.63-0.79)***	0.67 (0.60-0.75)***	0.62 (0.54-0.71)***
Migrants, non-Western	0.73 (0.67-0.79)***	0.72 (0.66-0.78)***	0.64 (0.58-0.70)***	0.60 (0.54-0.66)***	0.55 (0.49-0.62)***
18-24 years	1.53 (1.38-1.70)***		1.31 (1.17-1.45)***	1.30 (1.17-1.45)***	1.29 (1.16-1.43)***
25-29 years	1.00		1.00	1.00	1.00
30-34 years	0.95 (0.88-1.03)		1.00 (0.92-1.08)	1.00 (0.93-1.08)	1.01 (0.93-1.09)
35-40 years	0.88 (0.81-0.96)**		0.89 (0.81-0.97)*	0.88 (0.80-0.96)**	0.88 (0.81-0.97)**
Higher education	1.00		1.00	1.00	1.00
<High school/unknown	1.58 (1.46-1.71)***		1.71 (1.56-1.87)***	1.53 (1.40-1.68)***	1.53 (1.40-1.68)***
High school	1.54 (1.43-1.67)***		1.37 (1.26-1.49)***	1.32 (1.22-1.43)***	1.32 (1.22-1.43)***
Frequent primary care attendence	2.99 (2.71-3.30)***		2.51 (2.28-2.76)***	2.48 (2.25-2.72)***	2.46 (2.27-2.67)***
Not employed/studying	1.46 (1.36-1.57)***			1.45 (1.34-1.57)***	1.45 (1.34-1.57)***
Mother, perinatal * Western					1.15 (0.89-1.49)
Mother, > beyond perinatal * non-Western					1.15 (0.94-1.40)
Mother, perinatal*Western					1.27 (0.94-1.72)
Mother > perinatal*non-Western					1.29 (1.04-1.60)*

^*p*<0.10; *p<0.05, **p<0.01, ***p<0.001.

**Fig. 2:**
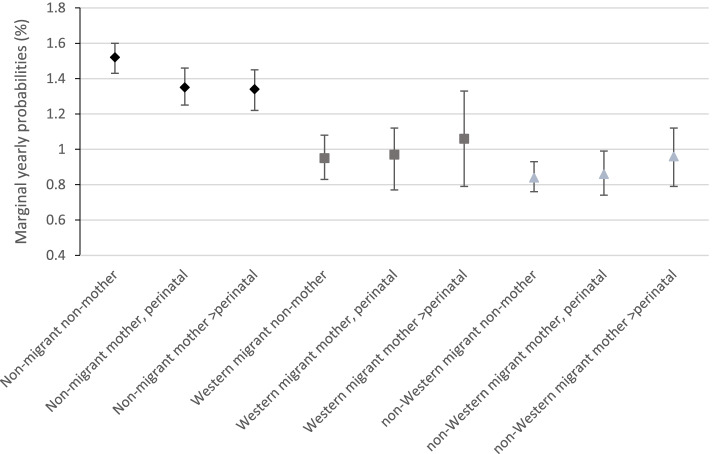
Marginal yearly probability of OPMH service use by migrant category and motherhood for married women

In the interaction analyses (model 4), the main effect of motherhood was significant for both perinatal and >perinatal mothers, indicating that among married non-migrant women, mothers had lower odds of using OPMH services than non-mothers. However, the interaction term indicated the relationship was significantly weaker for married non-Western migrant women. Although not significant for married Western migrant women, the odds ratios also indicated a weaker relationship. Plotting marginal yearly probabilities of OPMH service use by migrant category and motherhood (Fig. 2) indicated that the yearly probability of OPMH service use for both groups of migrants was similar regardless of motherhood. There was a slight difference for non-migrants; with non-migrant women without children having a greater probability than both groups of non-mothers. However, the 95% intervals were slightly overlapping.

We repeated analyses with the sample only including women who had been in Norway for at least five years and the results of the fully adjusted model and interaction model are shown in Table 3. In model 1, controlling for all covariates, mothers did not have significantly lower yearly odds of OPMH service use, though again, there was a tendency for perinatal mothers to have lower odds. Inclusion of the interaction in model 2 indicated that for married non-migrants, perinatal mothers had significantly lower odds of OPMH service use and mothers beyond the perinatal period tended to have lower odds. There was no significant interaction between motherhood and migrant category, suggesting that the relationship between motherhood and OPMH service use was not significantly different across migrant category for married women.

**Table 3:** Odds ratios and 95% confidence intervals for outpatient mental health service use^a^

	Model 1 (*n*=72998)	Model 2 (*n*=72998)
Non-mother	1.00	1.00
Mother, perinatal	0.92 (0.85-1.00)^	0.90 (0.82-0.99)*
Mother, > perinatal	0.94 (0.86-1.03)	0.90 (0.81-1.00)^
Non-migrants	1.00	1.00
Migrants, Western	0.75 (0.66-0.85)***	0.71 (0.61-0.84)***
Migrants, non-Western	0.62 (0.56-0.68)***	0.58 (0.51-0.66)***
18-24 years	1.27 (1.14-1.42)***	1.27 (1.14-1.42)***
25-29 years	1.00	1.00
30-34 years	0.98 (0.90-1.07)	0.99 (0.91-1.07)
35-40 years	0.87 (0.79-0.95)**	0.87 (0.79-0.96)**
Higher education	1.00	1.00
<High school/unknown	1.65 (1.49-1.82)***	1.65 (1.49-1.82)***
High school	1.36 (1.25-1.48)***	1.35 (1.25-1.47)***
Frequent primary care attendence	2.42 (2.19-2.67)***	2.41 (2.18-2.66)***
Not in employed/studying	1.56 (1.43-1.69)***	1.55 (1.43-1.67)***
Mother, perinatal * Western		1.08 (0.80-1.45)
Mother, > beyond perinatal * non-Western		1.11 (0.90-1.49)
Mother, perinatal* Western		1.14 (0.83-1.56)
Mother > perinatal* non-Western		1.20 (0.96-1.51)

^a^ Based on married women aged 18-40 and married migrants with ≥5 years in Norway; ^*p*<0.1, *p<0.05, **p<0.01, ***p<0.001.

### Labour market attachment, motherhood and OPMH service use

For characteristics of the sample of married women aged 25-40 years used in analyses with labour market attachment see Supplementary table 2. Table 4 shows the results of the fully adjusted model and three models with different interactions. We controlled for age, education level and frequent attendance in all models. Ongoing education was not significantly related to OPMH service use, so we excluded this in analyses.

**Table 4:** Odds ratios and 95% confidence intervals for outpatient mental health service use with interactions ^a,b^

	Model 1	Model 2	Model 3	Model 4
	(*n*=81908)	(*n*=81908)	(*n*=81908)	(*n*=81908)
Non-mother	1.00	1.00	1.00	1.00
Mother, perinatal	0.92 (0.85-1.04)^	0.86 (0.77-0.95)**	0.94 (0.86-1.02)	
Mother, > perinatal	0.92 (0.83-1.01)^	0.85 (0.75-0.95)**	0.93 (0.85-1.03)	
Mother				0.90 (0.82-0.99)*
Non-migrants	1.00	1.00	1.00	1.00
Migrants, Western	0.68 (0.61-0.77)***	0.62 (0.53-0.72)***	0.90 (0.78-1.02)	0.85 (0.71-1.02)*
Migrants, non-Western	0.61 (0.55-0.67)***	0.54 (0.48-0.62)***	0.80 (0.71-0.90)**	0.71 (0.61-0.84)***
No or weak labour market attachment	1.45 (1.33-1.57)**	1.44 (1.33-1.57)***	1.96 (1.77-2.16)***	2.01 (1.77-2.27)***
Mother, perintal * Western		1.27 (0.97-1.66)		
Mother, perintal * non-Western		1.29 (1.03-1.60)*		
Mother, >perintal* Western		1.30 (0.95-1.79)		
Mother, >perintal* non-Western		1.35 (1.06-1.72)*		
No/Weak labour market attachment* Western			0.41 (0.32-0.53)***	
No/Weak labour market attachment* non-Western			0.48 (0.39-0.58)***	
Non-mother*no/weak labour market attachment* Western				0.41 (0.30-0.55)***
Non-mother*no/weak labour market attachment* non-Western				0.49 (0.39-0.62)***
Mother*no/weak labour market attachment*non-migrants				0.91 (0.74-1.10)
Mother*no/weak labour market attachment* Western				0.51 (0.33-0.78)**
Mother*no/weak labour market attachment* non-Western				0.52 (0.39-0.71)***
Mother*stable labour market attachment*Western				1.19 (0.90-1.58)
Mother*stable labour market attachment* non-Western				1.28 (1.02-1.61)*

^a^ based on married women aged 25-40 years; ^b^ adjusted for age, education level and frequent primary healthcare attendance *p<0.05, **p<0.01, ***p<0.001.

In model 1, we found that overall, married women who became mothers tended to have lower odds of using OPMH services than married women who did not have children. This was for both perinatal mothers and mothers beyond the perinatal period. Again, we found a significant interaction between motherhood and migrant category (model 2). The relationship between motherhood and OPMH service use was significant for non-migrants but the interaction effect suggested this association was significantly weaker for non-Western migrant women. The interaction was in the same direction for Western migrant women, though it did not reach statistical significance. Marginal predicted probabilities indicated that the probability of OPMH service use was similar for married migrants regardless of motherhood status (Supplementary table 3). In model 3, there was an interaction between labour market attachment and migrant category. Noteworthy, the main effect of motherhood was not associated with lower odds of OPMH service use. Further, the difference in odds of OPMH service use between migrants from Western countries and non-migrants was not significant, indicating no difference in odds for these groups among married women with a stable attachment to the labour market. Although the main effect of labour market attachment was strong for non-migrants, the interaction term suggested it is far weaker for both groups of migrants. Marginal yearly probabilities indicated that the probability of OPMH service was not dependent on labour market attachment for married migrant women (Supplementary table 4).

**Fig. 3:**
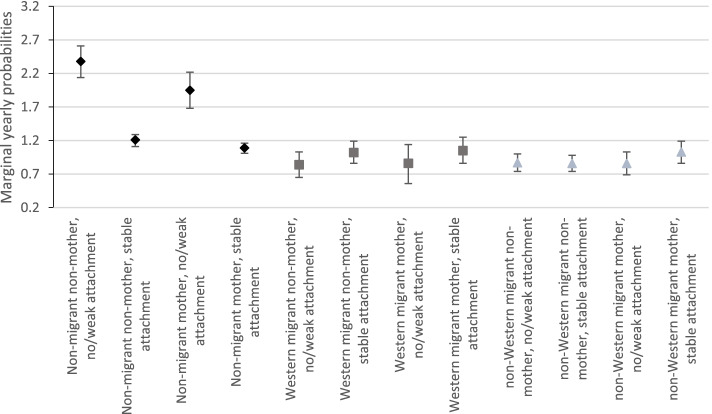
Marginal yearly probability of OPMH service use by migrant category, motherhood and labour market attachment

Finally, in model 4, we included a three-way interaction term. In this model, we combined both groups of mothers (perinatal and >perinatal) into one category to preserve statistical power and ease interpretation, since there was little difference in odds of OPMH for these groups. The main effects indicated that having no or a weak labour market attachment was associated with twice the yearly odds of OPMH service use for married non-migrant, non-mothers. Motherhood was associated with lower odds of OPMH service use for non-migrants with a stable labour market attachment. Further, married migrant non-mothers with a stable labour market attachment had significantly lower odds of using OPMH services compared with their non-migrant counterparts. The interaction term between mother*no/weak labour market attachment*non-migrants and mother*no/weak labour market attachment*Western migrants was not significant, suggesting that the relationship between labour market attachment and OPMH service use did not differ depending on motherhood for married non-migrants and married Western migrants. However, there were several other interactions. Plotted marginal yearly probabilities suggested that among married non-migrants, those with a stable attachment to the labour market had around half the probability of using OPMH services compared with non-migrants with no or weak attachment, for both mothers and non-mothers (Fig. 3). For both groups of migrants however, the yearly probability of OPMH service use was similar regardless of labour market attachment and motherhood.

Finally, we repeated the analyses on a sample including only migrants who had been in Norway at least five years together with non-migrants (see table 5). Our findings were similar indicating the robustness of the above findings, except that there was no significant difference in the strength of the relationship between motherhood and OPMH for either migrant group compared to non-migrants (model 2). Post-hoc marginal yearly probabilities for interactions in model 3 and model 4 are shown in Supplementary tables 5 and 6).

**Table 5:** Odds ratios and 95% confidence intervals for outpatient mental health service use with interactions ^a,b^

	Model 1	Model 2	Model 3	Model 4
	(*n*=65935)	(*n*=65935)	(*n*=65935)	(*n*=65935)
Non-mother	1.00	1.00	1.00	1.00
Mother, perinatal	0.91 (0.84-0.999)*	0.87 (0.78-0.96)**	0.93 (0.85-1.01)	
Mother, > perinatal	0.92 (0.83-1.02)^	0.87 (0.77-0.97)*	0.93 (0.84-1.03)	
Mother				0.91 (0.82-1.01)^
Non-migrants	1.00	1.00	1.00	1.00
Migrants, Western	0.75 (0.65-0.85)***	0.69 (0.58-0.82)***	0.93 (0.80-1.09)	0.91 (0.74-1.10)
Migrants, non-Western	0.63 (0.56-0.70)***	0.57 (0.50-0.66)***	0.81 (0.71-0.92)**	0.72 (0.61-0.86)***
No or weak labour market attachment	1.55 (1.42-1.69)***	1.54 (1.41-1.68)***	1.94 (1.75-2.15)***	2.00 (1.76-2.28)***
Mother, perintal * Western		1.21 (0.89-1.65)		
Mother, perintal * non-Western		1.24 (0.98-1.58)		
Mother, >perintal* Western		1.20 (0.86-1.68)		
Mother, >perintal*non-Western		1.26 (0.98-1.62)		
No/Weak labour market attachment* Western			0.46 (0.34-0.61)***	
No/Weak labour market attachment* non-Western			0.51 (0.41-0.63)***	
Non-mother*no/weak labour market attachment* Western				0.43 (0.30-0.63)***
Non-mother*no/weak labour market attachment* non-Western				0.54 (0.41-0.71)***
Mother*no/weak labour market attachment*non-migrants				0.90 (0.74-1.10)
Mother*no/weak labour market attachment* Western				0.51 (0.32-0.81)**
Mother*no/weak labour market attachment* non-Western				0.57 (0.41-0.79)**
Mother*stable labour market attachment* Western				1.08 (0.80-1.46)
Mother*stable labour market attachment* non-Western				1.28 (1.00-1.65)^^

^a^ based on married women aged 25-40 years and migrants with ≥5 years in Norway; ^b^ all models adjusted for age, education level and frequent primary healthcare attendance; ^*p*<0.10; ^^*p*=0.05, *p<0.05, **p<0.01, ***p<0.001.

## Discussion

In our study, we aimed to determine if married women who become mothers have higher or lower odds of mental disorder (using OPMH service use as a proxy) than married women who voluntarily or involuntarily do not have children. Our findings indicate that although married mothers may appear to have slightly lower odds of OPMH service use than married women who do not become mothers, this difference is mostly explained by age, education and workforce attachment in analyses with women aged 18-40 years. However, in analyses with married women aged 25-40 years and married migrants with at least five years in Norway, perinatal mothers had significantly lower odds of OPMH service use than married women who voluntarily or involuntarily do not have children. The difference in findings when we include younger women could be because previous research suggests that younger mothers tend to have poorer mental health [15, 52]. Nonetheless, although post-partum disorders can have serious consequences for the mother and child [3, 53], our findings suggest that the risk of developing a disorder is not greater at this stage, at least for married women, than it is for married women without children and may, in fact be lower for non-migrant women aged 25+ and migrant women with five or more years of residence.

Among married non-migrants, mothers both in and beyond the perinatal period have lower odds of OPMH service use than women without children. Previous studies considering mental disorder beyond the post-partum period found no difference in risk of depression or mental disorders in general between mother and non-mothers [7, 12] but neither of these studies accounted for mental health selection. Compared with non-migrants, we found the relationship between motherhood and OPMH use was weaker among married migrants, and significantly so for women from non-Western countries. Our post hoc analyses indicated that the yearly probability of service use was similar for both groups of migrants, regardless of motherhood status. It is possible that motherhood is associated with greater challenges for married migrant women, particularly those from non-Western countries, in terms of potential lack of practical support from extended family, challenges in bringing up children in a country with different values and practices, difficulties in navigating society, and the experience of discrimination [27, 28, 54]. Such additional stress may cancel out any protective aspect of becoming a mother on mental disorder. Since we found no significant difference in the relationship between motherhood and migrant category among married migrants who had been in Norway more than five years, it is possible that these challenges are less intense for women who have been in a new country for a longer period before having children.

On the other hand, previous research indicates that migrant women with stays of less than five years have particularly low utilisation of OPMH services [55]. Barriers to mental health service use are well-documented among migrants [56, 57] and it is likely that these barriers are greatest in the first few years after arrival [58]. Thus, the lack of relationship between motherhood and OPMH service use observed for married migrants in the main sample could indicate that OPMH service use is a poorer proxy for mental disorder among migrant women, especially those with shorter stays. Further, during the perinatal period, most women have more contact with healthcare services than prior to pregnancy [59, 60]. This allows greater opportunity for health professionals to identify mental health disorders and facilitate access to care. Thus, the lack of association between motherhood and OPMH service use for married migrant women may be because the unmet need for mental healthcare is greater among women without children, particularly those with shorter stays, than among women who become mothers. However, investigation of the gap between actual mental disorder and use of OPMH services among migrant women, both mothers and women without children, is required, as well as a closer investigation of the role of length of stay.

We also investigated if the association between labour market attachment and mental disorder differed for married migrants than for married non-migrants and by motherhood. Among married non-migrant women, a stable attachment to the labour market was associated with lower odds of OPMH service use. This association was the same regardless of motherhood. This supports the assumption that employment is beneficial, also for mother’s mental health [61]. However, labour market attachment was not significantly related to OPMH service use among either group of married migrant women, or dependent on motherhood status. Our findings were similar, even when we excluded women with less than five years in Norway at baseline (who are less likely to be in employment), demonstrating the robustness of this finding.

Migrant women, particularly those from non-Western countries have on average more job insecurity and poorer working conditions [38]. This could result in fewer mental health benefits compared with non-migrant women. We may therefore have expected a slightly weaker association between labour market attachment and OPMH service use for married non-Western women, especially among mothers, compared with married non-migrant women. Given how strong the relationship was among non-migrants, it is surprising that there was not even a weak association for either group of migrants. Survey data indicates that migrant women who are not in the workforce are more likely to report mental health problems than migrant women who are [62]. There are two potential reasons for this lack of relationship. The first relates to how well OPMH service use measures mental disorder among migrants with and without a stable labour market attachment. Married migrants who are in stable employment will presumably have more contact with Norwegian society, better language skills, more resources and possibly better health-literacy than migrants who do not. These attributes and skills may reduce barriers and lead to more appropriate help-seeking if they do experience a mental disorder [63, 64], particularly among women with longer stays. In support of this, our analyses indicated that married migrant women from Western countries who had a stable attachment to the labour force had similar odds of service use as married non-migrant women with a stable attachment. Married migrant women without a stable labour market attachment may, conversely, experience greater barriers and be less likely to seek help for mental disorder. Among married women with no or a weak labour market attachment, non-migrants had over twice the probability of using OPMH services as migrant women. Thus, there may be more unidentified mental disorder in migrant women with no stable attachment compared to women with a stable attachment to the labour market, resulting in no overall difference in service use between married migrant women with and without a stable labour market attachment.

The second potential explanation for the lack of observed difference in OPMH service use for married migrants by labour market attachment could be due to selection. A Norwegian study among mothers in the general population indicated that poorer mental health predicted subsequent workforce dropout, rather than non-workforce participation leading to poorer mental health [65]. This suggests that mothers with poor mental health are selected out of work. However, labour market attachment among migrant women, especially from Africa and Asia, is lower than for non-migrant women [41]. Mothers in particular may have a stronger preference to stay at home and care for one’s children [66]. Migrants may also struggle to enter the workforce due to lack of, or differences in, formal qualifications as well as discrimination [67, 68]. Thus, married migrant women, especially mothers, who do not work outside of the home may be a less selected group in terms of mental disorders than married non-migrant women.

### Strengths and limitations

This study has several strengths. The relationship between motherhood and mental disorder has not been researched much beyond the post-partum period for migrant women, and in our study, we were able to look at both the perinatal period and beyond. We were able to control for frequent primary care attendance for reasons not related to pregnancy, family-planning or mental health, as an indicator of general health. This is important as women without children report poorer general health than women in the general population [11]. We also attempted to account for mental health selection bias into motherhood by only including married women who had not used OPMH services in the two years prior to baseline. However, it is possible that there is a larger proportion of unidentified mental disorder among migrant women during these two years prior to inclusion, since they may experience greater barriers to care, particularly in the first few years after arrival. Further, the minimum of two years of residence in Norway will have led to an exclusion of some married migrant women who become mothers shortly after arrival.

A major limitation in this study is our inclusion of married women only. Non-migrant women are underrepresented in this study since a large proportion are unmarried when they become mothers [43]. Married non-migrant women without children may therefore be a more selected group in terms of health than married migrant women since, as indicated in an e-mail from Statistics Norway in 2021, childbearing prior to marriage is less common among migrant women from non-Western countries [69]. Thus, our findings might not be generalisable to unmarried non-migrant mothers. Further research should preferably include all women, but this will only be possible if information on their cohabiting status is available. Unfortunately, it was not possible in this study. We were also unable to account for whether women who did not become mothers were voluntarily or involuntarily childless. Involuntary childlessness is associated with poorer mental health [70]. Further, our measure of labour market attachment, based on income, does not give a nuanced view of women’s work situation in terms of the type of job, number of hours or extent of permanency. Such characteristics could have a different association with mental disorder. There may be other factors that differentiate between married women with and without children such as own number of siblings and urban residence [71]. Our non-migrant group is also diverse and includes 1) women born in Norway with Norwegian born parents, 2) women born in Norway with migrant parent(s) and 3) women born abroad with Norwegian born parent(s). Future studies could consider distinguishing between these different groups since they may have different marriage and childbearing patterns as well as different use of mental health services. Finally, we only had the child’s year, and not the date, of birth. In cases where OPMH service use and childbirth occur in the same year, we cannot determine which came first. However, exact date of birth was not available due to ethical reasons to preserve anonymity. Nonetheless, the life changing transition to motherhood, for many, begins during pregnancy and mental disorder during pregnancy is a strong predictor of post-partum disorder [72].

## Conclusions

In conclusion, this longitudinal study shows that the transition to motherhood among married women is not associated with higher odds of mental disorder, measured by use of OPMH services. Further, this also applies to mothers in the early childhood years. For married non-migrants, women who become mothers have lower yearly odds of using OPMH services than women who voluntarily or involuntarily do not have children. Labour market participation is associated with lower odds of service use for both mothers and non-mothers. Yet, a different picture emerges for married migrant women; among them, there was no significant association between motherhood and OPMH service use or labour market attachment and OPMH services. Future research should investigate if it is selection effects, barriers to healthcare, or both, that explain the lack of relationship for married migrant women. Married migrant women in general have lower OPMH service use compared with married non-migrant women, regardless of whether or not they become mothers. This might indicate the need to improve access to mental health care. Further research and targeted interventions for reducing barriers may be needed to bridge the gap between need for, and use of, mental health care among migrant women. Migrants with short lengths of stays and who do not have a stable attachment to the workforce may experience the greatest barriers.

## Supplementary information

**Additional file 1.**

**Additional file 2.**

**Additional file 3.**

## Data Availability

The datasets generated and analysed for the current study are not publicly available for data protection reasons. However, the data that support the findings of this study may be available from Statistics Norway and HELFO if ethical approval is granted.

## Abbreviations

OPMH: Outpatient mental health
EEA: European Economic Area
GP: General practitioner
