# Supplementary material for: The relationship between motherhood and use of mental health care services among married migrant and non-migrant women: a national register study
Source: BMC Psychiatry. 2022 Mar 21;22:211. doi: 10.1186/s12888-022-03848-9 (PMC8939178; doi:10.1186/s12888-022-03848-9)
Supplement: Supplementary file 1 — Additional file 1. [file 12888_2022_3848_MOESM1_ESM.docx]

| **Supplementary table 1: Overview of country composition of married migrant women categorised as Western and non-Western – total sample^1^** | |
| --- | --- |
| **Countries represented** | **Groups making up at least2% of sample** |
| **Migrants, Western (n=14410)** |  |
| Poland, Lithuania, Germany, Sweden, Romania, USA, Latvia, United Kingdom, Denmark, Bulgaria, Finland, Slovakia, The Netherlands, Estonia, France, Hungary, Spain, Canada, Australia, New Zealand, Greece, Ireland, Iceland, Italy, Croatia, Czech Republic, Cyprus, Faro Islands, Greenland, Liechtenstein, Luxemburg, Malta, Monaco, Portugal, Slovenia, Switzerland, Austria | Poland (37.2%), Lithuania (12.3%), Germany (7.2%), Sweden (6.9%), Romania (6.1%), USA (3.9%), Latvia, 3.2%), United Kingdom (2.4%), Denmark (2.1%) |
| **Migrants, non-Western (n=90195)** |  |
| The Philippines, Thailand, Russia, Pakistan, Iran, Iraq, Somalia, India, Vietnam, China, Afghanistan, Albania, Algeria, Angola, Argentina, Armenia, Azerbaijan, Bahrain, Bangladesh, Barbados, Belize, Benin, Bhutan, Bolivia, Bosnia-Herzegovina, Botswana, Brazil, Brunei, Burkina Faso, Burundi, Chile, Colombia, Costa Rica, Cuba, Curaçao, The United Emirates, The Dominican Republic, Djibouti, Ecuador, Egypt, El Salvador, Côte d’Ivoire, Eritrea, Ethiopia, Fiji, The Philippines, French Polynesia, Gambia, Georgia, Ghana, Grenada, Guatemala, Guinea, Guinea-Bissau, Guyana, Haiti, Honduras, Hong Kong, Belarus, India, Indonesia, Iraq, Iran, Israel, Jamaica, Japan, Yemen, Jordan, Cambodia, Cameroon, Cape Verde, Kazakhstan, Kenya, China, Kirgizstan, Comoros, Kongo, Kuwait, Laos, Lesotho, Lebanon, Liberia, Libya, Macao, Madagascar, Macedonia, Malawi, Malaysia, The Maldives, Mali, Morocco, Marshall Islands, Mauritius, Mexico, Moldova, Mongolia, Montenegro, Mozambique, Myanmar, Namibia, Nepal, Nicaragua, Niger, Nigeria, North Korea, Oman, Pakistan, Palestine, Panama, Papua Ny Guinea, Paraguay, Peru, Puerto Rico, Qatar, Russia, Rwanda, Reunion, Saint Lucia, Saudi-Arabia, Senegal, Serbia, Sierra Leonne, Singapore, Somalia, Sri Lanka, Sudan, Surinam, Syria, South Africa, South Korea, Tadzhikistan, Taiwan, Tanzania, Thailand, Togo, Tonga, Trinidad and Tobago, Tunisia, Turkmenistan, Turkey, Uganda, Ukraine, Uruguay, Uzbekistan, Venezuela, Vietnam, Zambia, Zimbabwe | The Philippines (12.6%), Thailand (11.3%), Russia (5.9%), Pakistan (4.6%), Iraq (4.2%), Iran (4.1%), China (4.0%), Somalia (3.9%), Vietnam (3.8%), India (3.7%), Brazil (3.0%), Bosnia-Herzegovina (2.8%), Turkey (2.6%), Ukraine (2.6%), Sri Lanka (2.4%), |
| ^1^ Country names are listed at time of migration | |
