# Supplementary material for: The relationship between motherhood and use of mental health care services among married migrant and non-migrant women: a national register study
Source: BMC Psychiatry. 2022 Mar 21;22:211. doi: 10.1186/s12888-022-03848-9 (PMC8939178; doi:10.1186/s12888-022-03848-9)
Supplement: Supplementary file 2 — Additional file 2. [file 12888_2022_3848_MOESM2_ESM.docx]

|  | | | | | | | |  |
| --- | --- | --- | --- | --- | --- | --- | --- | --- |
| **Supplementary table 2: Characteristics of the sample aged 25-40 years by migrant category^1^** | | | | | | | |  |
|  | **Total sample** | | | | | **Migrants > 5 years in Norway at baseline** | | |
|  | **Non-migrants (N=47057)** | **Migrants, Western (N=13832)** | | **Migrants, non-Western (N=21019)** | **Total (N=81908)** | **Migrants, Western (N=6674)** | **Migrants, non-Western (N=12204)** | **Total (with non-migrants) (N=65935)** |
| Mean (sd) years in study | 3.77 (1.85) | 2.91 (1.68) | | 3.14 (1.80) | 3.46 (1.85) | 4.12 (1.54) | 4.13 (1.67) | 3.88 (1.80) |
| Motherhood |  |  | |  |  |  |  |  |
| Non-mother | 19491 (42.42%) | 8597 (62.15%) | | 12836 (61.07%) | 40924 (49.96%) | 3189 (47.78%) | 6090 (49.90%) | 28770 (43.63%) |
| Mother, perinatal | 9537 (20.27%) | 2512 (18.16%) | | 3591 (17.08%) | 15640 (19.09%) | 1031 (15.45%) | 1898 (15.55%) | 12466 (18.91%) |
| Mother, > perinatal | 18029 (38.31%) | 2723 (1969%) | | 4592 (21.85%) | 25344 (30.94%) | 2454 (36.77%) | 4216 (34.55%) | 24699 (37.46%) |
| Used OPMH services | 2281 (4.85%) | 372 (2.69%) | | 623 (2.96%) | 3276 (4.00%) | 277 (4.15%) | 510 (4.18%) | 3068 (4.65%) |
| Age group |  |  | |  |  |  |  |  |
| 25-29 years | 12651 (26.88%) | 3173 (22.94%) | | 5670 (26.98%) | 21494 (26.24%) | 914 (13.69%) | 2707 (22.18%) | 16272 (24.68%) |
| 30-34 years | 18641 (39.61%) | 5162 (37.32%) | | 8021 (38.16%) | 31824 (38.85%) | 2766 (41.44%) | 4779 (39.16%) | 26186 (39.71%) |
| 35-40 years | 15765 (33.50%) | 5497 (39.74%) | | 7328 (34.86%) | 7328 (34.86%) | 2994 (44.86%) | 4718 (38.66%) | 23477 (35.61%) |
| Education |  |  | |  |  |  |  |  |
| <high school/unknown | 3607 (7.67%) | 4048 (29.27%) | | 9154 (43.55%) | 16809 (20.52%) | 1572 (23.55%) | 4711 (38.60%) | 9890 (15.00%) |
| High school | 9497 (20.18%) | 2794 (20.20%) | | 3264 (15.53%) | 15555 (18.99%) | 1309 (19.61%) | 2269 (18.59%) | 13075 (19.83%) |
| Higher | 33953 (72.15%) | 6990 (50.53%) | | 8601 (40.92%) | 49544 (60.49%) | 3793 (56.83%) | 5224 (42.81%) | 42970 (65.17%) |
| No or weak labour market attachment | 7120 (15.13%) | 5292 (38.26%) | | 10165 (48.36%) | 22577 (27.56%) | 1868 (27.99%) | 4383 (35.91%) | 13371 (20.28%) |
| Ongoing education | 4585 (9.74%) | 719 (5.20%) | | 2355 (11.20%) | 7659 (9.35%) | 470 (7.04%) | 1390 (11.39%) | 6445 (9.77%) |
| Frequent primary care attendence | 5785 (12.29%) | 610 (4.41%) | | 1781 (8.47%) | 8176 (9.98%) | 412 (6.17%) | 1351 (11.07%) | 7548 (11.45%) |
| Mean (sd) age of becoming a mother | 30.41 (3.54) | 30.94 (3.52) | | 30.51 (3.73) | 30.50 (3.58) | 31.19 (3.39) | 30.45 (3.64) | 30.49 (3.55) |
| ^1^ time-varying variables are shown at last year of inclusion | | |  |  |  |  |  |  |
