# Supplementary material for: The relationship between motherhood and use of mental health care services among married migrant and non-migrant women: a national register study
Source: BMC Psychiatry. 2022 Mar 21;22:211. doi: 10.1186/s12888-022-03848-9 (PMC8939178; doi:10.1186/s12888-022-03848-9)
Supplement: Supplementary file 3 — Additional file 3. [file 12888_2022_3848_MOESM3_ESM.docx]

| **Supplementary table 3: Predicted yearly probabilities (95% CIs^1^) among married women aged 25-40 years^2^** | |
| --- | --- |
| **Motherhood and migrant category** | **Yearly probability (%)** |
| Non-migrant non-mother | 1.46 (1.37-1.56) |
| Non-migrant mother, perinatal | 1.26 (1.14-1.37) |
| Non-migrant mother >perinatal | 1.24 (1.10-1.38) |
| Western migrant non-mother | 0.91 (0.79-1.03) |
| Western migrant mother, perinatal | 0.99 (0.78-1.20) |
| Western migrant mother >perinatal | 1.00 (0.73-0.13) |
| non-Western migrant non-mother | 0.80 (0.72-0.89) |
| non-Western migrant mother, perinatal | 0.88 (0.73-1.02) |
| non-Western migrant mother >perinatal | 0.91 (0.73-1.09) |

^1^ confidence interval ^2^corresponds with table 4, model 2

| **Supplementary table 4: Predicted yearly probabilities (95% CIs) among married women aged 25-40 years^1^** | |
| --- | --- |
| **Labour force attachment and migrant category** | **Yearly probability (%)** |
| Non-migrant, no or weak attachment | 2.21 (2.02-2.40) |
| Non-migrant, stable attachment | 1.15 (1.09-1.22) |
| Western migrant, no or weak attachment | 0.84 (0.68-1.00) |
| Western migrant, stable attachment | 1.04 (0.91-1.16) |
| non-Western migrant, no or weak attachment | 0.86 (0.76-0.97) |
| non-Western migrant, stable attachment | 0.93 (0.83-1.03) |

^1^ confidence interval ^2^corresponds with table 4, model 3

| **Supplementary table 5: Predicted yearly probabilities (95% CIs^1^) among married women, 25-40 years (migrants ≥5 years in Norway)^2^** | |
| --- | --- |
| **By labour force attachment and migrant category** | **Yearly probability (%)** |
| Non-migrant, no or weak attachment | 2.21 (2.01-2.42) |
| Non-migrant, stable attachment | 1.17 (1.10-1.24) |
| Western migrant, no or weak attachment | 0.97 (0.75-1.20) |
| Western migrant, stable attachment | 1.09 (0.94-1.25) |
| non-Western migrant, no or weak attachment | 0.93 (0.80-1.07) |
| non-Western migrant, stable attachment | 0.94 (0.83-1.05) |

^1^ confidence interval ^2^corresponds with table 5, model 3

| **Supplementary table 6: Predicted yearly probabilities (95% CIs^1^) among married women, 25-40 years (migrants ≥5 years in Norway)^2^** | |
| --- | --- |
| **Motherhood, labour market attachment and migrant category** | **Yearly probability (%)** |
| Non-migrant non-mother, no/weak attachment | 2.39 (2.13-2.63) |
| Non-migrant non-mother, stable attachment | 1.22 (1.13-1.31) |
| Non-migrant mother, no/weak attachment | 1.98 (1.69-2.23) |
| Non-migrant mother, stable attachment | 1.11 (1.02-1.20) |
| Western migrant non-mother, no/weak attachment | 0.95 (0.67-1.23) |
| Western migrant non-mother, stable attachment | 1.10 (0.90-1.30) |
| Western migrant mother, no/weak attachment | 1.02 (0.64-1.41) |
| Western migrant mother, stable attachment | 1.08 (0.86-1.31) |
| non-Western migrant non-mother, no/weak attachment | 0.95 (0.78-1.13) |
| non-Western migrant non-mother, stable attachment | 0.89 (0.75-1.02) |
| non-Western migrant mother, no/weak attachment | 0.92 (0.71-1.12) |
| non-Western migrant mother, stable attachment | 1.03 (0.86-1.21) |

^1^ confidence interval ^2^corresponds with table 5, model 4
